# Tribological Performance of Composites Reinforced with the Agricultural, Industrial and Post-Consumer Wastes: A Review

**DOI:** 10.3390/ma14081863

**Published:** 2021-04-09

**Authors:** Zuzanna Sydow, Mateusz Sydow, Łukasz Wojciechowski, Krzysztof Bieńczak

**Affiliations:** 1 Institute of Machines and Motor Vehicles (IMRiPS), Poznan University of Technology, 60965 Poznań, Poland; lukasz.wojciechowski@put.poznan.pl (Ł.W.); krzysztof.bienczak@put.poznan.pl (K.B.); 2 Łukasiewicz Research Network—Wood Technology Institute, 60654 Poznań, Poland; mateusz.sydow@itd.lukasiewicz.gov.pl

**Keywords:** waste, composite, polymer, metal, wear, coefficient of friction, tribology

## Abstract

Waste management is still one of the leading global challenges in the 21st century. From the European Union’s point of view, the Waste Framework Directive obliges businesses and households to recycle at least 55% of their municipal waste by 2025 and to reach 65% in 2035. Hence there is a great need to seek new solutions for the reuse of various waste materials. One of the most widely used wastes is their utilization as fillers or reinforcements in the metal- or polymer-based composites. The reuse of wastes for the production of tribological materials gives not only environmental benefits related to the transformation of waste into raw materials but also may improve the mechanical and tribological properties of such materials. Moreover, the use of waste reduces the production costs resulting from the lower price of filler materials and longer service life of developed products. The purpose of the current review is, therefore, aimed at the evaluation of the reuse of agricultural, industrial and postconsumer wastes as reinforcements in the composites used for tribological applications. The tribological performance (wear rate, coefficient of friction) of both monolithic and hybrid composites reinforced with waste materials was a particular subject of interest in this review.

## 1. Introduction

In the circular economy, responsible waste management and designing for recycling are increasingly often used slogans equated with sustainable development. Recently, the European Union has been intensively moving from linear economy to circular economy, which is assumed by keeping materials as long as possible in the loop and following the 3R approach: reduce, reuse and recycle. On 11 March 2020, the European Commission adopted a new Circular Economy Action Plan, which purpose is to “avoid waste altogether or transform it into high-quality secondary resources that benefit from a well-functioning market for secondary raw materials” [1]. Hence, a growing number of scientists pay great attention to designing this type of materials with the use of waste, such as agricultural (e.g., natural fibers), industrial (e.g., fly ash (FA), red mud (RM)) or postconsumer (e.g., polymer bags, clothes) waste materials. The properly prepared waste material may be used as a filler or reinforcement for metal or polymer-based composites aimed at use in various applications, e.g., as tribological materials directly related to the wear processes due to friction.

**Figure 1:**
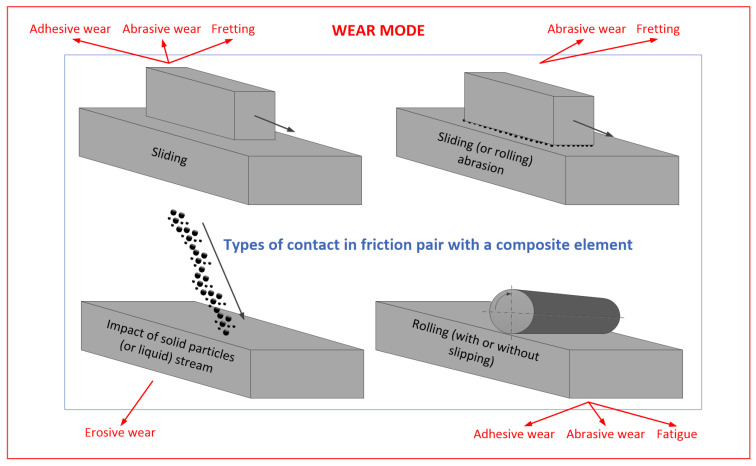
Types of contact between triboelements and the resulting wear modes.

Friction is a common phenomenon in nature and technology. It can be both a desirable and undesirable factor in a given situation. Friction is a disadvantageous phenomenon, e.g., during the movement of machine elements. Then it destroys cooperating, rubbing elements. This causes, for example, axles and bearings made of valuable materials to wear off. In addition, the friction causes a loss of energy to overcome the existing resistance. On the other hand, friction can be a useful or even necessary phenomenon, e.g., in stopping the body while it is in motion. Friction is essential for the proper functioning of brake pads, clutch, belt transmissions and many other elements. There are many ways to increase or decrease friction as needed, including the use of materials with appropriate tribological properties. To determine the suitability of the tribological material for a given application, the most important factors are the observed wear of material (the lowest wear is desirable) and the coefficient of friction (COF) (both low and high can be desirable). When describing wear, it is important to distinguish between the different tribological contacts depending on the relative motion of triboelements, as they lead to different wear mechanisms (see Figure 1). For example, sliding can lead to adhesive wear, abrasive wear and fretting, whereas rolling can lead to adhesive wear, abrasive wear and fatigue. Particle or liquid impacts lead to erosive wear of studied material. When considering small particles in the contact region, one can distinguish sliding (two-body) abrasion (when particles are blocked in the surface of one of the triboelements) or rolling (three-body) abrasion (when particles roll between two sliding bodies). Abrasion can be associated with abrasive wear and fretting. Wear can be quantitatively expressed as wear rate, usually defined as volume (or mass) loss per unit distance or specific wear rate, depending additionally on the applied load. Moreover, erosive wear depends largely on the applied impingement angle, i.e., an angle at which erodent particles impact sample(s) tested in the erosion wear test.

**Figure 2:**
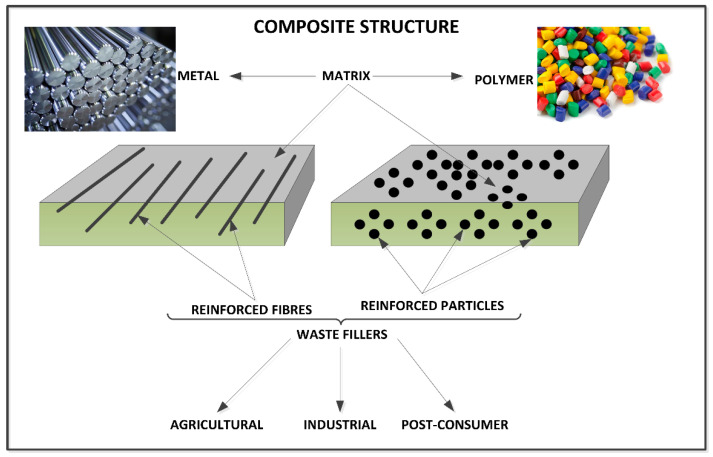
The general structure of metal- and polymer–matrix composites filled with various waste materials.

Materials for tribological applications can generally be divided into metals (and their alloys), ceramics, polymers and others. Among various metal materials used for tribological applications, aluminum and its alloys are one of the most commonly studied. However, these types of materials are characterized by low hardness and limited tribological properties, which, in turn, limit the possibilities of their application [2]. The most popular way to increase the strength of aluminum and its alloys is to add insoluble reinforcement to create a metal matrix composite. Both monolithic (single reinforcement) and hybrid (more than one reinforcement in composite) composites are developed, and in both cases, satisfactory results can be obtained in terms of tribological performance. In aluminum metal matrix composites, ceramic particles like carbides (e.g., SiC, B_4_C, TiC), oxides (e.g., Al_2_O_3_, MgO, ZrSiO_4_, ZrO_2_), borides (e.g., TiB_2_, AlB_2_) and nitrides (e.g., BN, AlN) are most often used as reinforcements. The addition of at least two reinforcements and thus the production of a hybrid composite is intended to enhance the mechanical and tribological properties compared to the materials reinforced with a single type of filler. The main disadvantage of this type of composites is often a reduction in ductility and an increase in brittleness of the composite [3]. On the other hand, many researchers primarily use polymer matrices to create composites reinforced with waste materials. Among these composites, one of the most commonly used is epoxy resin. Epoxy resin is resistant to many chemical agents (oils, greases), has high strength and good adhesion to the substrate, and exhibits higher resistance to cracking, environmental degradation and thermal decomposition compared to other thermosetting polymers [4]. However, there are also some obvious disadvantages of epoxy, such as its price, low impact and fracture resistance, as well as relatively high wear rate. Thus, to improve the mechanical performance and tribological properties, different fillers and reinforcements (e.g., nanotubes, SiO_2_, ZnS, MoS_2_, C-BN, H-BN, fullerene, graphite and graphene oxide) have been incorporated into epoxy in several investigations [5]. Hence, new reinforcements are sought to eliminate these drawbacks, e.g., waste-derived reinforcements (see Figure 2).

Recently, researchers have paid increasing attention to the use of waste materials as fillers/reinforcements for metal or polymer matrices to obtain composites exhibiting desirable tribological properties that would become an alternative to the traditionally used fillers. Such reuse of waste for tribological applications has several advantages: (i) environmental benefits by the transformation of waste into raw materials; (ii) an improvement of mechanical and tribological properties, as some researchers suggest treating such properly prepared materials as biosolid lubricants [6], (iii) economic benefits due to lower material production costs and longer service life (reduced wear rate); (iv) a reduction of the overall weight of the material, which is especially important in the case of metal materials used for tribological applications.

Literature reports indicate a relationship between tribological properties and mechanical properties. Therefore, the majority of publications investigate both of them. Taking into account the limited space of the article, this review focuses on the description of tribological properties without considering the mechanical properties. Experimental data were collected from scientific reports available until December 2020.

To the best of our knowledge, there is no review evaluating the tribological performance of metal and polymer-based composites reinforced with various agricultural, industrial and postconsumer wastes. In this article, a detailed review of polymer and metal–matrix composites filled with industrial, agricultural and postconsumer wastes is presented. This paper, containing four sections, is organized as follows: the first section deals with the agricultural waste materials used in metal and polymer matrix, the second section deals with the industrial waste materials used in metal and polymer matrix, the third section deals with postconsumer waste materials and the fourth section elaborate on the conclusions and the future scope of research.

## 2. Agricultural Waste Materials

### 2.1. Metal Matrix Composites

Metal matrix composites (usually based on aluminum matrix) are commonly used tribological materials. However, these types of matrices are often characterized by low hardness and limited tribological properties, which limits their possible application. Hence, there is still a need to create metal-based composites reinforced with appropriate agricultural fillers, enhancing both mechanical and tribological properties of the produced materials [2]. All of the studied metal matrix composites reinforced with agricultural waste materials are presented in Table 1.

#### 2.1.1. Ashes

An example of waste used in metal matrix composites is ashes obtained from various materials of agricultural origin. Rice husk is an abundantly available waste material generated in all rice-producing countries. Rice husk ash (RHA) is composed of various oxides, mainly silica (SiO_2_) and alumina (Al_2_O_3_) [55]. In the study by Arora and Sharma, RHA was incorporated into the aluminum matrix, and its properties were compared with that of a composite containing synthetic SiC reinforcement [41]. Reinforcements were used in the content of 0–8 wt %. All developed composites showed a lower sliding wear rate compared to the neat aluminum alloy, and the best results were obtained with maximal content of reinforcement, which was related to the increased material hardness. Although composites with SiC addition showed better antiwear properties than waste-based composites, the authors suggested that RHA-reinforced composites exhibited sufficient properties to be used as an alternative to SiC-reinforced composites. In another study, Prasad and Krishna examined the addition of RHA in the weight percentages of 0–8 wt % into aluminum alloy matrix [43]. They observed a decrease in sliding wear rate and COF with an increase in the content of RHA. However, the addition of 8 wt % RHA resulted in an increase of COF compared to the other variants for the two highest loads (39.22 N and 49.03 N). The addition of RHA into the aluminum matrix was also used by Shaikh et al. [42]. Researchers observed the increase in the sliding wear resistance of the prepared composites in all variants containing RHA, with the highest concentration (15 wt %) being less effective than the other two studied contents (5 and 10 wt %). In addition to monolithic composites (where only one filler is used), hybrid composites (where more than one filler/additive is used) can also be produced. An example of a hybrid composite with two agricultural waste fillers can be Mg-Al alloy with RHA and eggshell as reinforcements in 0–10 wt % [59]. The authors showed the optimal reinforcement content of 7.5 wt % with the lowest sliding wear loss and COF. In another study, the same authors additionally determined the wear mechanisms related to the studied composites. The dominant mechanisms were defined as delamination, abrasion and oxidation in mild wear regime, as well as melting and plastic deformation in ultra-severe wear regime [58]. Comparison of the tribological properties of aluminum alloy reinforced with FA and RHA (0–12 wt % of each waste used separately) were made by Krushna et al. [56]. The authors showed that the addition of each waste improved the antiwear properties compared to the neat matrix, wherein the lowest sliding wear was observed for the FA-reinforced composite. In another study, RHA and FA were simultaneously introduced into the A356 aluminum matrix in the following variants: (i) alloy/5% RHA–5% FA, (ii) alloy/10% RHA–10% FA, and (iii) alloy/12.5% RHA–12.5% FA. The best tribological performance—a 50% reduction in sliding wear and COF—was observed for the alloy/10% RHA–10% FA variant [57]. In another research, the same authors confirmed superior tribological properties of alloy/10% RHA–10% FA compared to other variants (the highest wear resistance and the lowest COF) [55]. In addition, they found that among many examined factors, the reinforcement particle size and the weight fraction are of the greatest importance for the properties of the composite.

Bean pod ash nanoparticles, which are considered low-density, cheap and available in huge quantities of agricultural waste, were added to the Al-Cu-Mg matrix [52]. It was determined that the wear rate was significantly lower for the reinforced composites than for the neat matrix, and an increase in filler content from 0 to 4 wt % improved the wear resistance. The authors also observed an increase in the COF of bean pod ash-containing composites compared to the alloy matrix.

Another agricultural waste filler used as a reinforcement in aluminum matrices is coconut shell ash. Raju et al. observed the lowest sliding wear in the case of the composite reinforced with 15 wt % of the waste (the studied contents ranged from 0–15 wt %) [50]. In turn, COF decreased with the increase in the weight percentage of the reinforcement.

Bannaravuri and Birru investigated the tribological properties of the Al-Cu alloy matrix reinforced with bamboo leaf ash (0–6 wt %) [44]. Among the variants, the best sliding wear resistance was demonstrated by composite filled with 4 wt % of the waste. The same authors, in another literature report, used the Taguchi approach to determine the influence of the tested parameters on the wear rate, specific wear rate and COF of aluminum-bamboo leaf ash composites [45]. Taguchi method is often used to select the most important control factors influencing the tribological performance of studied material, minimizing the number of experiments. The authors showed that the lowest wear rate was observed at 20 N applied load, 4 wt % bamboo leaf ash and 3.5 m/s sliding velocities.

Suleiman et al. used melon shell ash at 0–20 wt % as reinforcement material in Al-Si alloy [53]. They revealed that the higher content of melon shell ash was, the lower the sliding wear rate was observed. They also showed that wear rate was closely related to hardness, which also increased with increasing waste content.

Significant reduction in wear rate for composites reinforced with sugarcane bagasse ash (0–12 wt %) was indicated by Shnakar et al. [9]. The authors observed that the increase in waste content resulted in a decrease in sliding wear rate and a decrease in COF. In another study, bagasse ash waste (0–4 wt %), SiC (0–3 wt %) and magnesium (0–1 wt %) were used as additives to create aluminum/SiC/bagasse ash hybrid composite [10]. The authors observed that the sliding wear rate reached the lowest value for the variants with maximal 4 wt % bagasse ash. Moreover, an introduction of SiC caused a further gradual reduction in wear rate.

Dry sliding friction of aluminum-based composites reinforced with palm kernel shell ash produced using friction stir processing was studied by Fono-Tamo et al. [54]. The authors showed that COFs of newly developed composites were within the range exhibited by most aluminum matrix composites used in industry but can be characterized as a more eco-friendly material.

Other researchers used straw ash (agricultural waste, 5 wt %) and met coke ash (industrial waste, 5 wt %) as waste fillers in hybrid composites [60]. Additional studied non-waste fillers were mortar ash (5 wt %) and nanofibrillated composite (5 wt %). The authors examined the individual and combined effect of reinforcements on aluminum alloy. All studied composites showed better antiwear properties than neat aluminum alloy. The best sliding wear resistance among composites was observed for alloy-nanofibrillated composite and alloy-mortar ash-met coke ash, while the worst for alloy-mortar ash-nanofibrillated composite-met coke ash (excluding neat Al6064 alloy).

Coconut shell ash (0–8 wt %) and other additional additives (graphite and magnesium) were used to reinforce the aluminum matrix and improve its sliding wear resistance, as demonstrated by Panda et al. [49]. It was observed that all types of composites showed better antiwear properties compared to aluminum matrix. However, the best antiwear properties were achieved in the case of variant reinforced with 4 wt % of waste. For this variant, however, the highest fluctuations in COF were observed, which was explained by the interactions of hard particles with disc material, which also corresponds to superior wear resistance properties. Coconut shell ash (3 wt % in an aluminum matrix) was also investigated by Subramaniam et al., but in this case, the additional modifier was boron carbide (B_4_C) (0–12 wt %) [51]. The authors showed that the B_4_C and coconut shell ash additives significantly improved the antiwear properties compared to the neat matrix and alloy-coconut shell ash composite.

Boron carbide was also used for cow dung ash (CDA) reinforced aluminum composites, as shown in Manikandan and Arjunan [3]. Both additives were used in weight percentages of 0–7.5 wt %. It was observed that the sliding wear rate gradually decreased for all composites containing reinforcement due to less plastic deformation. COF also decreased for reinforced composites compared to alloy due to the release of soft CDA during the wear process, which acted as a solid lubricant. Wear rate and COF decreased in the following order: 2.5 wt % B_4_C + 7.5 wt % CDA; 5 wt % B_4_C + 5 wt % CDA; 7.5 wt % B_4_C + 2.5 wt % CDA.

#### 2.1.2. Shells

Shells are the protective outer layers usually created by the animals—either to protect the eggs (eggshells) or animals themselves (a calcareous exoskeleton, which protects the soft parts of an animal, e.g., snail shells). Shells are primarily composed of calcium carbonate and, to some extent, other minor components. Chicken eggshell wastes were used to reinforce aluminum matrix as described by several publications. Hayajneh et al. observed an increase in wear resistance by 65% for the composites reinforced with 3–4 wt % of eggshells. The lowest value of COF was obtained for a variant with 4 wt % of filler. However, in the case of higher waste contents, a negative effect was observed, which was explained by the increase in porosity and agglomeration of particles [46]. In another study, Dwiwedi et al. investigated the influence of eggshell reinforcement (0–10 wt %) on the composites based on the Al6061 aluminum alloy matrix [47]. The lowest wear rate was observed for the variants with maximal filler content (10 wt %). Furthermore, Dwivedi et al. incorporated waste carbonized eggshell (0–12.5 wt %) and silicon carbide (SiC, 0–12.5 wt %) into AA2014 aluminum alloy to create green composites with superior tribological properties [48]. The authors showed that regardless of the waste content in the matrix, the wear rate remained similar for all of the studied variants.

Enyi et al. investigated Zn-ZnO-snail shell particles composite coating on mild steel [61]. Waste snail shell particles were used from 0 to 25 g for each sample. The authors observed that the addition of snail shell particles decreased the sliding wear rate of composite coatings, and the best antiwear properties were achieved when 15 g of snail shells were added.

### 2.2. Polymer Matrix Composites

Other types of matrices commonly used as tribology material are polymers. Among the polymer matrices, epoxy resin is the most commonly used for composites containing agricultural waste material. Other polymer matrices described in the literature include polypropylene (PP), polyethylene (PE), polyester, polyvinyl chloride and natural rubber. All of the studied polymer matrix composites reinforced with agricultural waste materials are presented in Table 1.

#### 2.2.1. Plant Fibers or Their Derivatives

Natural fibers are a promising source of filler for composites due to many advantages, such as biodegradable/renewable nature, low weight resulted from low-density, wide availability, low-cost compared to biodegradable synthetic polymers, especially taking into account the fact that they are often a waste product [62].

*Jatropha curcas* L. fibers were incorporated into the epoxy matrix in the form of whole seeds cake, seed shells and seed kernels cake at weight percentages up to 20 wt % [8]. It was observed that all types of produced composites significantly increased the wear resistance up of 69% to 82% against neat epoxy, with the best results achieved with the whole seed cake filler. The results of improving wear properties using *Jatropha curcas* L. fibers as a filler at 0–40 wt % in the epoxy matrix were also obtained by Shivamurthy et al. [6]. The authors observed a reduction in the specific wear rate and COF of developed composites compared to neat epoxy, with the lowest values of specific wear rate and COF achieved for a variant with 40 wt %. of filler. On the other hand, in the study of three-body abrasive wear, Hrabě and Müller noted that epoxy matrix composites with *Jatropha curcas* L. oil cake (0–30 wt %) showed higher weight loss compared to the neat matrix when free abrasive fraction F60 was used [7]. However, when other free abrasives (F400) were used, composites performed comparably with the neat epoxy.

Ruggiero et al. prepared epoxy-based composites reinforced with date stones or date flesh (from *Phoenix dactylifera*) at weight percentages of 0–10% [17]. It was shown that an increase in the content of hard particles of date stones resulted in an increase of abrasive wear resistance (up to 11%). On the other hand, the composites filled with particles originating from the date flesh caused a decrease in wear resistance.

Valášek et al. investigated tribological properties of composite composed of the empty bunch fiber from palm oil production and epoxy matrix [21]. The authors used the above-mentioned fibers of different lengths and weight percentages (0–10 wt %) and alternatively applied a chemical treatment using 6% NaOH. Authors indicated that in terms of tribological properties, the additive of natural filler did not relate considerably to two-body abrasion resistance. The addition of fibers did not exhibit a positive effect but also did not show a negative effect, which is a desirable result in the context of replacing epoxy matrix content with cheap and ecological waste material.

The tribological properties of composites consisting of palm kernel fibers, a waste material from the palm oil extraction process, and epoxy matrix have been investigated by several authors. Mat Tahir et al. determined the influence of sliding distance and temperature on epoxy composites reinforced with palm kernel activated carbon (70 wt %), which combines carbon and residual oils (natural lubricant) properties [11]. The authors observed that with an increase of sliding distance, the specific wear rate slightly increase, while COF does not change significantly up to 90 °C. The study demonstrated that below 90 °C, palm kernel activated carbon-epoxy composite poses unique self-lubricating properties, which distinguishes it from other agricultural-based and synthetic composites. Additionally, the influence of contact pressure and sliding speed on wear rate and COF of epoxy composites reinforced with palm kernel activated carbon (60 wt %) was investigated by Mahmud et al. [12]. The authors showed that there is a critical limit of contact pressure, above which the wear rate and COF increase drastically regardless of the sliding speed. Prakash et al. tested nano-activated carbon material obtained from waste Arhar stalks as a filler for the epoxy-based composites [14]. Different contents of filler (up to 3 wt %) were used in the study, and the minimum erosion and abrasive wear rate were observed for 2 wt % of filler, which gave significantly greater wear resistance compared to the unreinforced matrix.

Several types of thermoset matrices (orthoptic polyester resin, isophthalic polyester resin, and vinyl ester resin) reinforced with fiber bundles (10 wt %) obtained from *Musaceae rachis* were used by Correa et al. to compare the effect of fiber size on wear resistance of the produced composites [28]. Authors indicated that, in general, all types of composites were characterized by lower specific mass loss compared with the neat matrix. However, the best antiwear resistance was achieved for vinyl ester resin and 287 μm size of fibers.

The effect of *Citrus limetta* peel fiber (after conversion into the particulate form) as a reinforcement (15 wt %) in an epoxy matrix on antiwear properties was studied by Sharma et al. [20]. The authors showed that all of the tested composites achieved lower wear loss and COF values compared to the matrix itself. Additionally, the best tribological performance was observed for composites with the finest particulate size of the used filler.

Tribological properties of PP composites reinforced with wood flour waste (0–55 wt %) were studied by Ibrahim et al. [23]. The obtained results indicated that the developed composites exhibit improved tribological properties (lower COFs and lower abrasion rates), which resulted from the robust interfacial adhesion of components. The lowest specific wear rate was obtained for composite filled with 15 wt % of wood flour.

Polyvinyl chloride was used as a matrix to create composites based on olive stone flour waste (0–50 wt %) [30]. Authors observed that weight loss increased with the increasing weight percentage of filler—for variants with 50 wt % of filler it increased by 95% compared to the neat polymer. The authors explained the effect of lowering wear resistance by increasing the stiffness of composites containing olive stone flour, as well as increasing the roughness resulting from the protrusion of filler particles.

An example of a hybrid composite based on the epoxy matrix is a composite filled with glass fibers/pawpaw stem fibers [13]. The authors examined layers of fibers in linear and network structures with 0–15 wt % of combined reinforcement. The lowest weight loss was observed at 3 wt % for linear and 15 wt % for network composites. Other authors added Tasar silk waste (0–14 wt %) to an epoxy matrix with jute reinforcement (non-waste material), thus creating a hybrid material with the improved tribological properties [19]. The lowest specific wear rate was observed for the concentration of 12 wt % of the waste at a sliding velocity of 5.3 m/s. Kumar and Prasad incorporated *Pongamia pinata* seed cake waste called Pongamia oil cake (0–6 wt %) into basalt epoxy composites [15]. The obtained result suggested that the sliding wear resistance was improved by the addition of Pongamia oil cake, wherein the best antiwear properties were observed in the case of composite reinforced with 6 wt % of waste filler. A hybrid composite made of a polyester matrix and various waste fillers—soap nuts seeds, *Aegle marmelos*, and *Terminalia chebula* (each filler in 0–12 wt %)—were studied by Prasad et al. [25]. The authors showed that with the increase of wastes contents, the abrasive wear rate of composites also increased. The polyester matrix reinforced with waste palm fronds and mango’s dry leaves (separately) at different weight percentages (0–50 wt %) were studied by Ibrahim [27]. All of the waste materials increased the wear resistance of composites but had a different effect on the COF, as it increased with an increase in the content of palm fronds particles. On the other hand, COF decreased with increasing content of soft particles of mango’s dry leaves. Hence, according to the authors, composites with palm fronds can be used for high friction and low wear rate applications, e.g., brake pads, while with mango’s dry leaves (especially soft) for low COF and low wear rate applications, as solid lubricants.

Many authors, apart from the matrix and waste fillers, select appropriate abrasive additives to create an abrasive tool, e.g., asbestos-free brake pads. Often, the composition includes both a polymer part and a metal part (aluminum), but due to the fact that there is more of the polymer part, these composites are included in the chapter dealing with polymer matrices. Then, obviously, for such materials, low wear is expected, but on the other hand, a relatively high COF is needed. Examples of such materials are composites investigated by Bakry et al. containing fibers: sugar bars, corn, and palm firewood used at 10–25 wt % (as agricultural wastes). Furthermore, other ingredients, i.e., silica, commercial carbon, barium sulfate, metallic powder (10–30 wt %) and phenol–formaldehyde resin, were incorporated into the composite [36]. In general, the addition of waste fibers caused a slight increase in COF and a drastic decrease in wear as the content of waste increased. The highest COF was obtained for composites containing 25 wt % of sugar bar fibers and 30 wt % of iron, while the lowest wear was observed for composites containing 20–25 wt % of sugar bar fibers and 20 wt % of copper. The asbestos-free friction materials with natural waste ingredients (bagasse fiber and banana peel) were investigated by Amirjan [37]. The composition of the produced composites based on waste bagasse fiber and banana peel (0–10 wt %); alumina (4 and 8 wt %), rock wool, carbon fiber, glass fiber, steel fiber, silica, magnesium oxide, graphite, brass powder, vermiculite, phenolic resin, calcium carbonate and barite. The authors observed that a sample with 5 wt % of bagasse fiber and 4 wt % of alumina exhibited the lowest values of specific wear rate and COF. Waste corn stalk as reinforced material in friction composites was investigated by Ma et al. [38]. The authors created a composite containing 0–8 wt % of corn stalk fiber and constant contents of other ingredients: vermiculite powder, compound mineral fibers, calcium carbonate, coke, graphite, friction powder, zirconium silicate, alumina, barium sulfate, zinc stearate and phenolic resin. A positive effect of the presence of reinforcement on tribological properties was observed for each variant, while the best performing specimen with the lowest wear rate was demonstrated by a sample containing 6 wt % of corn stalk fibers. Another example of waste used to create friction material is cow dung fibers (0–8 wt %), which were compared to corn stalk fibers (0–8 wt %) [39]. Other additives included: graphite, coke, zinc stearate, vermiculite powder, calcium carbonate, zirconium silicate, alumina, compound mineral fibers, friction powder, phenolic resin and barium sulfate. The authors observed a positive effect of the addition of cow dung fibers resulting in higher wear resistance and COF compared to composites without waste. Additionally, composites containing cow dung fibers showed more stable COFs and higher wear resistance compared to composites containing corn stalk fibers. Coconut natural fiber was used in friction composites by Cracium et al. [40]. The authors used 5 and 10 wt % of coconut fiber and other ingredients: aluminum, graphite, zirconia oxide, silicon carbide, titanium oxide and phenolic resin. The authors indicated that both composites containing 5 and 10 wt % coconut fiber show almost similar properties and could be a candidate for the fabrication of asbestos-free brake pads.

#### 2.2.2. Ashes and Shells

Polyvinyl chloride matrix was reinforced with RHA as presented by Chand et al. [31]. Prior to composite development, RHA (0–40 wt %) was modified by a compatibilizing agent, i.e., maleic anhydride. The authors observed that the appropriate treatment of the filler improved the abrasive wear resistance of a composite. However, the content of the filler must be kept at the lowest of the tested contents—10 wt %. Higher amounts of RHA caused an inadequate wetting and inhomogeneous dispersion of filler in matrix, which in turn contributed to the deterioration of mechanical and tribological properties. Other authors used RHA waste (10 and 20 wt %) and CaCO_3_ (10 wt %) as reinforcements to create monolithic and hybrid composites based on PP matrix [22]. Research has shown that CaCO_3_ can be successfully replaced with RHA to create composites with optimal tribological properties for, e.g., yarn for the textile industry. Oladele et al. used calcinated particles of African land snail shells to reinforce epoxy matrix and tested variants with various particles size and 0–10 wt % of filler [4]. The authors observed that the addition of 10 wt % with <75 μm size particulates resulted in a significant reduction (>90%) of abrasion wear of the epoxy matrix.

An interesting solution is the use of both a filler from waste material and a matrix in the form of recycled polymer. An example of such a solution is the study by Oladele et al., wherein authors used snail shell waste (0–15 wt %) as a filler and recycled PP (obtained from the manufacturer in the form of damaged plastic chairs and tables) [29]. The obtained results showed that due to the fact that snail shell particulates are rich in hard and rigid CaCO_3_ phase, this kind of reinforcement significantly lowers the wear index. The lowest wear index was obtained for 15 wt % of waste shells, which exhibited a 52% higher abrasive wear resistance than the neat matrix. A similar approach for the use of snail shell waste (0–15 wt %) with recycled polymer was described by Atuanya and Aigbodion [32]. However, the authors used a different matrix—recycled PE. The authors showed that COF increased, and wear rate decreased with an increase in the content of snail shell particulates. Additionally, the results indicated that the addition of the smallest shell particulates (125 μm) showed the best antiwear properties.

Composites composed of high-density polyethylene (HDPE) as a matrix and poultry eggshell-derived hydroxyapatite (0–40 wt %) as a filler were studied by Oladele et al. [33]. The authors showed a significant increase in wear resistance of the prepared composites compared to the neat HDPE. Moreover, the addition of 40 wt % filler caused a maximal wear rate reduction (by 125%) than the control HDPE. Natural rubber matrix reinforced with different sources of CaCO_3_ was studied by El Mogy et al. [35]. The authors used either eggshell or fishbone waste fillers (constant 30 wt %) and compared them to commercial calcium carbonate. All of the tested composites showed better abrasive wear resistance compared to neat natural rubber, while the composite containing commercial CaCO_3_ was characterized by a lower abrasion loss than composites containing waste fillers.

The polyphenylene sulfide matrix was used to produce particulate mussel shell waste-reinforced composites (0–10 wt %) [34]. The authors showed that all composites show better adhesive wear resistance compared to neat matrix, with the best results obtained for the composites filled with the lowest weight percentage of waste, i.e., 1 wt %. Further increasing the filler content resulted in an increase in adhesive wear volume.

#### 2.2.3. Other Agricultural Wastes

Another example of a waste material incorporated into a composite is bovine hair fibers (added at 0–20 wt %) tested by Agbeboh et al. [26]. All tested composites showed significantly higher abrasive wear resistance compared to non-reinforced unsaturated polyester matrix. However, the highest enhancement of tribological performance was obtained for the composite containing 10 wt % of filler. Biological waste—short human hair (0–6 wt %) or/and glass microspheres were incorporated into epoxy matrix to create a hybrid composite, as shown in Prasad Nanda and Satapathy et al. [18]. The authors showed an improvement in the wear performance of reinforced epoxy composites (minimal wear for composites filled with 6 wt % of waste), with the wear resistance properties being further improved with the addition of glass microspheres. The abrasive wear resistance of bovine femora-reinforced epoxy composites with 0–20 wt % of filler was determined by Olajide et al. [16]. The authors indicated that the 20 wt % of waste significantly improved the antiwear properties of the composite.

Nutraceutical coriander seed spent, and congo red dye adsorbed onto nutraceutical coriander seed spent were used as fillers (0–50 wt %) for PP-based composites by Taqui et al. [24]. The study showed that the three-body abrasive wear increased with an increase in filler content, applied load and sliding distance.

## 3. Industrial Waste Materials

### 3.1. Metal Matrix Composites

Aluminum-based matrices are most commonly reinforced with various industrial waste materials and evaluated concerning their tribological performance. All of the studied metal matrix composites reinforced with industrial waste materials are presented in Table 2.

Fly ash (FA)—being one of the most common industrial waste materials—is a coal combustion byproduct composed of various oxide particulates (dominated by silicon dioxide SiO_2_, aluminum oxide Al_2_O_3_, ferric oxide Fe_2_O_3_, calcium oxide CaO, magnesium oxide MgO, potassium oxide K_2_O and sodium oxide Na_2_O) [108]. These oxides are the main components of coal-bearing rocks (and rocks in general). One of the first publications focused on the utilization of FA in metal matrices was an article by Uthayakumar et al. [107]. The authors carried out multifactor-based experiments on a dry sliding wear system of stir-cast aluminum alloy 6351 with 5, 10, and 15 wt % FA reinforced composites. The authors observed that at lower loads, the sliding wear, specific wear rate, and COF were decreasing with the increasing percentage of FA. However, with the increase of the load, wear of the composite was increasing with an increase of FA content. In anther study, Dinaharan et al. [109] estimated the wear rate of AA6061 aluminum reinforced with FA (added in the amount of 0–18 vol %) based on the experiments using pin-on-disc wear apparatus. The obtained results showed that with increasing percentage of FA in the composite, wear rate decreased from 411 × 10^−5^ mm^3^/m at 0 vol % to 203 × 10^−5^ mm^3^/m at 18 vol %. Simultaneously, the authors observed the improvement in microhardness of the developed aluminum matrix composites. Rani Panda et al. [108] investigated the influence of FA (15 vol %) addition on the wear resistance of aluminum-silicon metal matrix. The authors obtained the best results for variants reinforced with plasma-treated FA (caused by the in situ conversion of SiO_2_ to hard SiC particles). In the study by Krushna et al. [56], Al6061 aluminum alloy was reinforced with up to 12 wt % of FA. The results showed that the specific wear rate of the FA-based composite was always lower than pure metal alloy and alloy reinforced with identical wt % of RHA. One of the FA components—fly ash cenospheres—was utilized by Bera and Acharya, who investigated its influence (0–12.5 wt %) on abrasive wear behavior of LM6 aluminum alloy composite [111]. Their results showed that, independently of the applied load and sliding distance, the composite with 10 wt % of fly ash cenospheres was superior to other variants in terms of the quantified wear rate. Furthermore, bottom ash—the heavier ash fraction obtained in coal combustion plants that does not rise up with flue gases—was investigated in terms of its application in forming aluminum composites (0–10 wt %) [118]. The obtained results suggested that the values of wear rate and COFs were similar among all of the studied variants (including pure aluminum) up to 40 N of load. Exceeding the 40 N load, the composites containing 5 and 10 wt % of bottom ash exhibited higher wear rates and lower COFs than pure aluminum. The authors indicated, however, that adhesive wear was dominant for pure aluminum, whereas abrasive wear was most important for the formed composites. FA as a waste product is often used as a co-reinforcement combined with other conventional and waste additives. Patil et al. investigated the impact of combined FA/SiC on the 7075-T651 aluminum alloy-based hybrid composites [110]. Both reinforcements were added in different SiC:FA ratios (from 60:40 to 90:10) and different volume percentages of combined additives (from 4 to 12 vol %). The lowest wear rate was observed for samples with a SiC/FA ratio of 75:25 and a total reinforcement volume of 8%. At the same time, maximal microhardness was achieved for the ratio of 90:10 and a total reinforcement volume of 12%. The authors indicated that the improvement in microhardness and wear behavior was especially evident in samples with maximum SiC content and for ratios with less than 20% of FA in the mixture of reinforcements. In the study by Kumar et al., two industrial waste materials—FA and RM—were used simultaneously to prepare an as-cast A356 aluminum alloy-based hybrid surface composites (with equal volume percentages of both wastes reaching 9%) using friction stir processing [114]. The results suggested that the presence of reinforcements improved the microhardness and wear resistance of the aluminum alloy.

Red mud (RM) is an alkaline industrial waste generated in vast amounts during the production of alumina. Its red color is caused by the main constituent of RM—iron oxide (III) Fe_2_O_3_, which makes up ca. 30–50% of its mass [113,114]. The utilization of RM for the production of metal matrix composites with enhanced tribological properties was an object of interest of two studies. Singla et al. demonstrated that in the case of 6061 aluminum alloy-based composites RM waste can successfully replace expensive conventional reinforcement materials, such as SiC and alumina [113]. Although the wear rate of RM-reinforced composites was slightly lower than SiC-reinforced composites, the values were comparable to those of Al_2_O_3_-reinforced composites. The lowest wear rate for RM-reinforced composites was obtained for 7.5 wt % of RM. In another study, Devi Chinta et al. investigated the aluminum-based hybrid composites containing constant weight percentages of tungsten carbide (4%) and increasing percentages of RM (2–6%) [112]. The obtained results confirmed that the highest wear resistance could be obtained for hybrid-composites containing 6 wt % of RM (regardless of the used particle size).

Ceramic wastes can also be a useful source of reinforcing materials. Waste porcelain ceramic particulate was used with the combination of constant weight percentage of B_4_C to produce AA7075 hybrid aluminum composite [124]. The authors showed a reduction in wear loss and values of COF with porcelain content increments up to a critical value (12 wt %), after which it began to increase. In another study, Zheng et al. demonstrated that aluminum-based hybrid composite containing both SiC (10 wt %) and ceramic waste (20 wt %) exhibited the highest COF (higher than pure aluminum alloy and SiC only reinforced composite) comparable with those obtained for conventional brake disc material [123]. The wear rate was, however, much lower than the conventional brake disc regardless of the applied load.

Another interesting group of industrial wastes are slags—byproducts generating during metal smelting. Slags contain various metal oxides (mainly Fe_2_O_3_ and silicon dioxide SiO_2_). Prabhakaran and Arul characterized Lm6 aluminum alloy reinforced with copper slag (up to 10 wt %) [115]. Their results indicated that the lowest wear rate and the highest Vickers hardness were obtained for composites with maximal used percentage weight of copper slag. In another study, Sridhar Raja et al. investigated the tribological performance of A356 aluminum alloy composite reinforced with waste steel slag (0–12 wt %) [119]. The authors observed that the weight loss due to wearing was reduced gradually with an increasing weight percentage of steel slag particles and was minimal for composite with 12 wt % of the waste.

The processing of quarry rocks generates wet grinder stone dust particles that can be used to reinforce aluminum metal matrix composites, as shown in Xavier and Suresh [120]. The authors showed that the highest studied amount of waste addition (20 wt %) was characterized by a maximal wear resistance and hardness. Another interesting waste obtained during the processing of rocks is crushed rock sand, which is produced while crushing the rock into pieces for construction purposes. Ashok Kumar and Devaraju investigated the high-temperature wear behavior of 7075 aluminum alloy-based composites reinforced with crushed rock sand and SiC (separately and together) [117]. The best results were obtained for a hybrid composite containing a maximal amount of SiC (i.e., 6 wt %) and 3 wt % of crushed rock sand (the maximal percentage was 6 wt %). However, the same variant also exhibited the highest COF, which is desired in terms of its application as a material used in the brake drum. Granite particulate—a waste generated while cutting granite stones—was an object of interest of Satyanarayana et al., who investigated its impact on the tribological performance of the aluminum-silicon alloy A356 (also with co-addition of graphite) [121]. The obtained results suggested that either reinforcement with sole granite (2 wt %) or with a combination of granite and graphite (4 and 2 wt %, respectively) contribute to a decrease in the value of COF as compared to neat A356 alloy. In another study, a limestone slurry powder (also a waste from the stone cutting industry) was used to reinforce the aluminum-magnesium-silicon alloy matrix [116]. The authors observed a decrease in COFs for samples reinforced with the limestone slurry powder, with the optimal weight percentage of waste being 12% (the maximal studied content of waste was 16 wt %).

Krishnan et al. study was focused not only on the utilization of the waste as a reinforcement but also as a matrix [122]. The authors used scrap aluminum as a matrix and spent alumina catalyst from the oil industry as a reinforcement (5 wt %). The produced composites were compared with other variants containing either AlSi7Mg alloy or alumina Al_2_O_3_. The authors observed the highest weight loss in the abrasive test for a composite consisting of both wastes, whereas the lowest weight loss was demonstrated by a composite composed of scrap aluminum waste and alumina.

Although the majority of the studies were focused on pure aluminum or aluminum-based alloys, also composites based on other metals were tested concerning their tribological performance. For example, in the study by Zikin et al., recycled tungsten carbide (WC) and cobalt (Co) hard metal powder (originating from hard metal scarp) was used as a coating of Castolin nickel-based powder and compared with the nickel-based reference hardfacing consisting of 40 vol % of tungsten carbide [125]. The results showed that the produced coating was less resistant to abrasive wear (by a factor of two) than conventionally used reference containing tungsten carbide. In another study, silicon bronze alloy was reinforced with marble dust particulates (0–10 wt %) [126]. The experiment exhibited that depending on the studied tribological parameter (i.e., sliding velocity or normal load), the values of specific wear rate and COF differed among the variants. The lowest specific rate (while studying sliding velocity) reached the lowest value for the composite with 10 wt % of marble dust. On the other hand, while investigating the normal load influence on specific wear rate, the lowest wear rate was observed for the composite with 2.5 wt % of marble dust. Marble dust (1.5–6 wt %) reinforced copper alloy was also used by Rajak et al. [127]. The authors observed that all composites were characterized by lower wear loss against different sliding velocities compared to copper alloy without reinforcement. However, the best antiwear resistance was observed for 4.5 wt % of reinforcement. In addition, COF was the highest in the case of a sample with 4.5 wt % of reinforcement and the lowest for copper alloy without marble dust. Thus, the authors suggested the composites with 4.5 wt % of marble dust for bearing applications.

### 3.2. Polymer Matrix Composites

Industrial wastes are more commonly used as reinforcements for polymer matrices. All of the studied polymer matrix composites reinforced with industrial waste materials are presented in Table 2.

FA and fly ash cenospheres were extensively studied for their application as additives to polyester, polyethylene, epoxy resin or nylon matrices. Bishoyee et al. evaluated the erosive wear rate of composites produced from polyester resin, E-glass fibers (50 wt %) and cenospheres (0–20 wt %) [94]. The authors indicated that the variants with maximal (20 wt %) content of fly ash cenospheres exhibited the lowest erosion rate (regardless of the applied impingement angle). In another study, Chand et al. examined organosilane modified cenospheres (10–20 wt %) as a filler for an HDPE [102]. Abrasive wear tests showed that the lowest specific wear rate, but also the highest impact strength, were obtained for composites reinforced with 10 wt % of organosilane treated fly ash cenospheres. The authors suggested that the observed increasing wear rate of composites with the increasing weight percentage of cenospheres is probably caused by the rapid chipping of the particles and matrix at the interface. The hybrid composites made of epoxy resin, bamboo fiber (33 wt %) and fly ash cenospheres (0–6 wt %) were investigated by Jena et al. [85]. The results of erosion wear tests showed that the lowest value of erosion wear rate was observed for the composite with maximal (6 wt %) content of fly ash cenospheres. Two industrial wastes—FA and granite powder—were the subject of the study carried out by Ray et al., who used both wastes separately (0–15 wt %) to reinforce composite made of polyester resin (45–60 wt %) and glass fiber (constant 40 wt %) [95]. Although the addition of both reinforcements significantly reduced the erosion rate of the composites, the variant with 15 wt % FA filler turned out to be the most resistant to erosion. The same authors also investigated identical composites concerning their abrasive wear resistance [93]. In contrast to their previous results focusing on erosion resistance, the composites filled with 15 wt % granite powder showed the best abrasion resistance.

RM waste was often studied as reinforcement in polymer-based composites. Biswas and Satapathy evaluated the erosion rate of hybrid bamboo–epoxy and E-glass-epoxy (the content of either bamboo or E-glass fibers was 50 wt %) composites filled with RM in various weight percentages (0–20 wt %) [67,68]. Their results showed that the increase of the RM content improved the erosion resistance of the composites, with the lowest erosion rate obtained for bamboo–epoxy composite with 20 wt % of RM. A sliding wear test for epoxy-based composites (either homogenous or graded) reinforced with 0–20 wt % of RM was carried out in Siddhartha et al. [69]. The authors indicated that the lowest specific wear rate and the lowest COF were exhibited by homogenous composites with 10–20 wt % of RM reinforcement. Another polymer matrix—unsaturated polyester resin—reinforced with nanoparticles of RM (0–4 wt %) was studied by Suresh and Sudhakara [96]. Based on the results of the sliding wear test, the authors observed that regardless of the applied sliding speed, the lowest wear rates and COFs were obtained for nanocomposites filled with a maximal 4 wt % of RM. In another study, Richard et al. also evaluated the dry sliding wear properties of the nanocomposites made of unsaturated polyester resin and various size nanoparticles of RM (0–2.5 wt %) [97]. The authors obtained the lowest specific wear rate for nanocomposites reinforced with the highest content (2.5 wt %) of 110 nm-sized RM particles (i.e., particles with the smallest studied size). A hybrid composite composed of unsaturated polyester resin, pineapple fiber (non-waste material) and RM (10–20 wt %) was evaluated concerning its either sliding wear or erosion resistance by Sundarakannan et al. [98]. The results obtained by the authors indicated that with an increasing weight percentage of RM reinforcement, the sliding wear rate of composites decreases, whereas the erosion wear rate increases, suggesting that the potential utilization of the fabricated composite may depend on its potential industrial application.

Other wastes generated during the metallurgical processing of metal ores are slags and sludges produced during iron and steel making. Linz–Donawitz (LD) slag is generated from an LD converter during the production of steel and is mainly composed of CaO, Fe, and SiO_2_. Blast furnace (BF) slag is solid waste generated from blast furnace and contains various oxides, such as SiO_2_, CaO, Fe_2_O_3_, MgO, Al_2_O_3_. The other waste is LD sludge solid waste generated during the cleaning of flue gas emerging from the LD converter. It mainly contains FeO, Fe_2_O_3_ and CaO [75]. LD slag was an object of interest of several publications of Pati and Satapathy and Pati et al. [70,71,87]. As shown by Pati and Satapathy, the reinforcement of the composites with LD slag improved the erosion wear resistance of the developed epoxy-based or hybrid glass fiber-epoxy (20 wt % of glass fiber) composites with the best erosion resistance observed for maximal weight percentage of LD slag (either 22.5 or 30 wt %). In the study by Pati et al., hybrid glass fiber-propylene composites filled with up to 22.5 wt % of reinforcement were evaluated concerning their erosion wear. The authors observed that LD slag content is a significant control factor for minimizing the erosion rate of the produced composites. Numerous publications of Purohit and Satapathy studied the influence of LD slag, LD sludge and BF slag on the sliding wear and erosion wear of the composites based on epoxy resin matrix [72,73,74,75]. The obtained results indicated that with increasing content of filler, both erosion wear- and sliding wear resistance of the composites increases. Additionally, their comparative studies showed that either sliding- or erosion wear rate reached minimal values for composites filled with LD sludge, while for composites filled with LD slag and BF slag, the results were comparable. In another study, Erdoğan et al. carried out a study comparing the BF slag, converter slag and ferrochromium slag (30 wt % in each case) as a material used to reinforce epoxy-based composites [76]. The obtained results showed that the produced slag reinforced composites generally exhibited similar or better tribological properties than alumina reinforced composites, while the highest sliding abrasion resistance was observed for composite filled with BF slag. Hybrid composites prepared by reinforcement of PP matrix with BF slag (0–30 wt %) and (alternatively) short glass fibers (0–20 wt %) were investigated by Padhi and Satapathy [88]. The authors demonstrated that the highest erosion wear resistance was obtained for composites filled with a maximal 30 wt % of BF slag (regardless of the addition of glass fibers). The waste obtained at the final refining step of a hydrometallurgical zinc plant was evaluated as a reinforcement for difunctional epoxy monomer-based resin [86]. The results of the study indicated that the lowest wear rate could be obtained for composites with 30 vol % of waste (the maximal studied volume of waste was 50%) and for the largest studied particle size (i.e., larger than 208 μm). Another waste generated during various processes of iron and steel forming is an iron scale composed mainly (ca. 96%) of iron oxide (III) Fe_2_O_3_. This waste was utilized as a filler (0–20 wt %) in a propylene matrix by Erdogan et al. [91]. The authors showed that the lowest COF and volume loss was exhibited by the composite variant containing 5 wt % of iron scale. The aluminum smelting process produces residue waste called white aluminum dross containing mostly alumina (Al_2_O_3_). In a study by Samat et al., this type of waste was used as a reinforcement in PP-based composites with various weight percentages of the filler (0–40 wt %) [89]. The authors indicated that the lowest wear rate was exhibited by a composite variant containing the highest amount of the dross (i.e., 40 wt %). The same aluminum dross was used to produce micro- and nanosized alumina, which was further utilized as a filler (0–7 wt %) in PP-based composites [92]. The lowest wear rate was exhibited by the composite reinforced with 7 wt % of nanosized alumina, but each of the tested variants performed better than pure PP.

Other potentially useful waste materials are byproducts obtained during the processing of different types of rocks. Marble dust waste is generated during the processing of the marble rocks and is composed mainly of CaCO_3_, MgCO_3_, CaO, MgO and other oxides (SiO_2_, Al_2_O_3,_ etc.) [81]. Choudhary et al. investigated the influence of marble dust (0–30 wt %) and E-glass fiber mat (10 layers) on the tribological performance of epoxy-based composites [81]. Composites with the highest marble dust content exhibited the lowest erosion rate regardless of the applied impingement angle and impact velocity. In their two literature reports, Nayak et al. determined the sliding wear behavior of hybrid composites made of unsaturated polyester resin, E-glass fiber mat (10-layers, 40 wt %) and waste marble dust (0–16 wt %) [99,100]. The authors demonstrated that the highest sliding wear resistance was observed for the composites with maximal waste marble dust weight percentage (16 wt %). Granite powder—a solid waste generated from the stone processing industry—was evaluated in terms of its potential use as a reinforcement (0–15 wt %) in a hybrid composite containing epoxy resin and glass fiber (40 wt %) [82]. The performed erosion wear tests showed that 10 wt % of filler addition was optimal concerning erosion resistance of the studied materials. Slate powder, obtained from slate rock tailings, is a mineral waste material that was used to produce hybrid composites made of phenolic resin (10–35 wt %), glass fiber (10 wt %), alumina (7.5 wt %) and graphite (7.5 wt %) [103]. These composite formulations containing 40–65 wt % of slate powder were compared to similar composites containing barite (65 wt %). The authors observed that the best wear performance was exhibited by the variant containing 40 wt % of slate powder, which was slightly below the level of the composite produced with the use of barite. Iron mud, a major solid waste produced in iron mining and ore processing, was an object of interest in numerous studies performed by Pani et al. [63,64,65,66]. This waste is composed mainly of iron oxide (III) Fe_2_O_3_, aluminum oxide Al_2_O_3_ and silicon oxide SiO_2_ and became a serious threat to the soil environment due to its long-term storage. The authors demonstrated that glass fiber-epoxy composites (50 wt % of glass fiber) reinforced with 0–20 wt % of iron-mud waste could be potentially used as tribological materials. The highest abrasive wear resistance was obtained for composites reinforced with a maximal 20 wt % of iron-mud. However, regarding the erosion wear resistance, no clear relationship between the iron-mud content and erosion wear was observed as it changed unevenly with impingement angle and erodent velocity.

Boron-containing waste generated during borax production was an object of interest of two studies carried out by Uygunoglu et al. [77,78]. This waste containing similar amounts of B_2_O_3_, SiO_2_, CaO and MgO was used up to 50 or 66 wt % to reinforce the epoxy resin matrix. Abrasive wear tests performed in the first study showed that the composite with maximal content of boron-containing waste (50 wt %) exhibited the lowest abrasive wear rate and COF. The second study, however, indicated that the wear length of the composites slightly increased with increasing the filler content up to 33 wt %, which was explained by the poor interfacial bonding with the epoxy matrix.

In a study by Lin and Schlarb, waste carbon fibers were used as a reinforcement (10 wt %) in polyether ether ketone (PEEK) polymer matrix, which was also filled with solid lubricant (graphite), ZnSO_4_ and TiO_2_ [106]. The obtained results revealed that composites reinforced with recycled carbon fibers exhibited similar tribological properties to the variants filled with virgin carbon fibers. In another study, Aslan et al. investigated the tribological performance of PP-based composites containing sisal fibers (up to 30 vol %, non-waste material), waste carbon fibers (up to 27 vol %) and waste E-glass fibers (up to 21 vol %) [90]. The lowest values of COF were obtained for composites not containing sisal fibers, especially the composites reinforced with 27 vol % of waste carbon fibers. In the study of Acikbas and Yaman, waste glass fibers (5–20 wt %) and waste wall tile (40–55 wt %, a waste from wall tile factory) were used to reinforce epoxy resin matrix [84]. The authors demonstrated that the lowest wear rate was exhibited by the fine particle composite containing 55 wt % of waste wall tile and 5 wt % of waste glass fibers.

Another waste material that caught the attention of researchers is red brick dust, a powder formed from deformed bricks in the process of their manufacturing. Pati developed a hybrid epoxy composite containing 15 wt % of short E-glass fibers and various amounts of red brick dust (0–30 wt %). The obtained materials were evaluated in erosion wear tests and exhibited better erosion wear resistance than epoxy-glass fiber composites, with the lowest erosion wear for composites filled with 30 wt % of red brick dust.

Coal mine overburden waste was an object of interest in the study performed by Das et al. [83]. This waste material was mixed with epoxy resin matrix in various proportions (0–40 wt %) and evaluated concerning its sliding wear resistance. The obtained results indicated that the highest content of waste material (40 wt %) caused a maximal wear resistance among the studied variants.

Ruggiero et al. utilized waste glass beads—a waste material originating from glass blasting—to produce epoxy-based composites with improved tribological properties [79]. Comparing the composites with the control and those filled with non-waste glass powder, the lowest values of COFs and the lowest wear were observed for variants containing 5–20 vol % of the largest size of waste particulate.

Another material—carbon obtained from the pyrolysis of polymer wastes—was used in the study by Myalski et al. to reinforce polyamide (PA) thermoplastic composites [105]. The 10 wt % addition of this modified waste material, however, lead to an increase in COF as compared to neat PA variant (although the differences were not statistically significant), suggesting its poor performance as a reinforcement.

One of the oldest studies dealing with waste management for tribological purposes was a publication of Xiang and Tao, who studied mechanical and tribological properties of composites produced from polytetrafluoroethylene (PTFE) and PTFE waste (20 wt %) generated during the manufacturing of various PTFE products [104]. The authors indicated that the COF increased with the addition of PTFE waste, but at the same time, a significant decrease in wear rate was observed. It was also observed that the addition of alumina nanoparticles (15 wt %) further improve the tribological performance of the produced materials.

## 4. Postconsumer Waste Materials

In addition to typically used agricultural and industrial wastes, postconsumer waste is also a valuable source of filler materials. Postconsumer waste is a material discarded after its use by consumers and includes, e.g., packaging, broken devices and items, clothes, etc. All of the studied composites reinforced with postconsumer waste materials are presented in Table 3.

An example of such waste material is carbon nanomaterials obtained from waste PE bags. Khan et al. developed epoxy-based composites reinforced with 0–3 wt % of such filler and compared them with composites containing commercial carbon nanotubes as well as neat epoxy [128]. The produced composites containing carbon nanofiller from PE bags showed better tribological properties than the neat epoxy matrix and even commercial carbon nanotubes. The specific wear rate of composites filled with carbon nanomaterials was reduced by 57%, and COF was reduced by 70% as compared to neat matrix.

Khan et al. utilized carbon fibers synthesized from waste clothes and then alternatively modified by oxygen plasma treatment [129]. Fillers in the amount of 0–3 wt % were added to the epoxy matrix. It was found that both the addition of treated and untreated carbon fibers improved tribological properties—wear and COF decreased, with the best results obtained for plasma-treated (200 W) composites containing 3 wt % of filler—in this case, the COF decreased by 84%, while specific wear rate by 76% as compared to neat epoxy.

Several researchers focused on waste tire dust (WTD) transformed into a form of small particles/dust, which are introduced into polymer matrices with appropriate additives. This is done to, e.g., create new friction materials used in brake pads (in these cases, an increase in COF is desirable). An example is a composite described by Mutlu et al., which consists of the following components: WTD (5–15 wt %), phenolic resin, Cu particles, alumina, graphite, brass particles, cashew and barite [132]. The highest COF was obtained for the sample containing 15 wt % of WTD. Additionally, the samples with the highest COF were characterized by the highest wear rate. The high porosity of WTD was determined in brake pads, which is an important factor for friction materials. Ruzaidi et al. focused on palm slag (0–40 wt %) friction composites additionally filled with WTD (0–40 wt %) to improve the hardness and slide wear properties [135]. The friction material additionally contained polyester resin, metal fiber, alumina and graphite. Based on the results, it was concluded that composites with the ratio of palm slag/WTD equal to 30/10 wt % and containing the largest size of filler particles (>600 μm) exhibit a moderate hardness with better wear resistance than commercial brake pads. Composites reinforced with waste tire rubber powder (0–40 wt %) and PP were investigated by Sivaraos et al. [131]. The lowest COF value was obtained for the neat PP matrix, while the introduction of filler content increased the value of COF (the highest COF was obtained for composite reinforced with 40 wt % of waste material). On the other hand, in the study by Adesina et al., it was observed that the addition of WTD reinforcement (0–20 wt %) into epoxy resin caused a reduction in COF [5]. However, it was also observed that for the variants with up to 10 wt % of WTD, the wear resistance was improved by over 70%.

A new wear-resistant composite material with zeolite-reinforced polytetrafluoroethylene (PTFE) modified by waste engine oils was investigated by Petrova et al. [134]. It is an unusual approach since usually waste materials are used as a filler, while here, the authors used waste engine oils as modifiers of PTFE. The developed composites not only exhibited significantly reduced wear (due to the presence of zeolites) but also stabilized COF at a constant low level. This situation would not be possible in the case of zeolite-reinforced composites without any modifiers.

Sabaa and Fahad utilized window glass and porcelain tile waste (both from the construction site) as abrasives in epoxy resin matrix [130]. The authors observed that porcelain composites showed better properties than glass composites for abrasive tools (low wear rate and high hardness), with the best results being obtained for grit size of 150 μm. Olesik et al. used glass powder (0–30 wt %) obtained from laminated car glass waste as reinforcement in a low-density PE matrix [133]. The authors obtained 3D-printed LDPE-based composites with the improved wear resistance resulted from the formation of a sliding film on the composite surface. The lowest specific wear rate was obtained for composites filled with 15 wt % of glass powder.

Copper surface composite filled with E-waste cathode ray tube glass (5–15 wt %, obtained by wrecking the obsolete color television) and boron nitride were produced by friction stir processing by Gopal and Kavimani et al. [136]. The authors observed that the addition of waste reinforcement caused an increase in sliding wear resistance and a slight increase in COF during sliding. Therefore, it was suggested that composites containing this type of waste could be utilized in wear-resistant applications.

## 5. Future Perspectives

This review indicated that industrial and agricultural wastes are mainly used for tribological applications. Among the agricultural wastes, the most frequently described wastes are various natural fibers of plant origin (in a polymer matrix) and RHA (in the metal matrix), whereas among industrial wastes, FA, RM, and various slags and sludges, are the most commonly used. It seems that at least some of them have a high potential to reduce frictional resistance and wear.

Aluminum alloy metal matrix composites can be a good example here. The specific configuration of their properties, such as high mechanical strength and corrosion resistance with low weight, predispose them as a hypothetical material for manufacturing of many frictionally working parts like pistons, plane bearings, sliders, brake rotors, etc. Unfortunately, the problem is often the high production costs of parts based on such composites. That is why low-cost materials with comparable characteristics are in demand. In our review, we indicate applications in which the use of RHA as a reinforcing filler for a composite with an AlSi10Mg alloy matrix significantly improved its wear resistance [137]. Another example would be the use of RHA as a filler for the composite with the AA6351 matrix. In this case, only a slight decrease in wear resistance was observed compared to the reference material. Therefore, it is possible to use agricultural waste fillers for composites with an aluminum matrix without adversely affecting their tribological properties.

Even greater possibilities apply to composites with typically plastic matrixes. There are also great possibilities in the application of waste fillers to compose the structure of composites with typically polymer matrixes. The results of the scratch tests presented in Ariharan et al. [22] indicate that the resistance to wear of an RHA-reinforced PP composite exhibits the same wear resistance as the reference material (PP composite without RHA). Therefore, it can be assumed that PP-RHA composites will be suitable for tribological applications of classic PP composites. In practice, these can be parts operating under light to medium-heavy sliding friction conditions, e.g., plain bearings or gaskets and inner and outer races of rolling bearings (balls and rollers are steel or glass). Another example would be the use of snail shells (as a filler) in PE matrix composites. In this case, it was observed that the addition of waste filler increased the hardness and wear resistance compared to the reference composite (PE composite without snail shells) [32]. PE composites are widely used in modern technics. Therefore, their versions with waste fillers may prove to be an interesting alternative for less responsible applications, e.g., low-speed bearings, runners for bottling in filling lines, chain guides in plate conveyors, etc. The responsible application of these composites as camshafts in engines, valve trains, or biomedical bearing material (joint prostheses) can also be considered. PA composites are another widely used group of polymeric materials in which waste fillers can be used. Biocarbon obtained from the pyrolysis of polymer wastes can be an example of it. In the investigation carried out in Myalski et al., the wear of the PA composite with this filler was characterized by much lower wear in the sliding test on cast iron than standard PA6 and other PA composites [105]. This “paves the way” for PA composites with waste fillers for the production of plain bearings as well as gears. Recycled carbon fibers can also be used as a filler for PEEK-based composites. According to Lin and Schlarb, this type of composite is characterized by excellent friction and wear performance [106]. PEEK is one of the polymers with the best tribological properties, including high operating temperature, resistance to most chemical reagents, and suitability for work under high contact stresses. The disadvantage is the high production cost. Therefore, the use of waste filler can be economically advantageous without losing strength properties. The application of such composites can be wide—from the previously mentioned bearings and gears to friction pairs in aviation and aerospace, working at temperatures exceeding even 200 °C.

Although the heavy industry is the largest producer of potentially hazardous waste material, the agricultural byproducts seem to be an interesting alternative since they can deliver various materials and substances that can be reused. For example, the total production value of food waste in the world is estimated at $1 trillion, whereas about one-third of food is wasted globally [138]. Fruit seeds seem a unique waste material that cannot only be used to produce bioactive compounds [139,140] but also offers a potentially valuable fiber. Although there are some studies dealing with the disposal of fruit seeds (e.g., from *Jatropha curcas* L. or *Phoenix dactylifera* L.) for tribological applications [8,17], the highest seeds waste is generated from other, more popular fruits [139]. Poland is one of the biggest fruit producers in Europe (behind Spain, Italy, France and Greece), and at the same time is the second biggest sour cherry producer in the world [141]. As sour cherries are mostly being processed and not consumed fresh, there is a high need to utilize the generated seeds for various applications, e.g., for tribological materials.

Postconsumer wastes seem to also be promising, albeit still not so popular, filler materials. Although this review indicates the potential possibilities of their use in tribological materials, it is still a research area to be expanded, especially regarding the fact that the disposal of textile products or plastics at the end of their life is a huge environmental problem. The global market of composites made from polymers and textile fibers is expected to increase by 40% from 2014 to 2020, which results from many desirable properties, such as high-strength values and corrosion resistance [142]. However, these types of materials require a special pretreatment, including the process of recovery of fibers from textile materials by mechanical, chemical, or biochemical methods. These methods should also be optimized to be as eco-friendly and material-efficient as possible. To gain a maximal environmental effect, there is still a need for the development and selection of the optimal treatment methods for all types of wastes. However, to select the best waste material for a particular application and the best methods of its treatment, various environmental tools, such as Life Cycle Assessment, should be used. It is also crucial to utilize the most abundant materials in the first place (e.g., FA) still considering their possible migration from the composite (and possible toxic or ecotoxic effect), but also to reuse wastes available locally (e.g., sour cherries seeds in Poland) to avoid transport-related emissions. Only taking these parameters into account will allow for the production of truly green materials exhibiting desirable tribological properties.

## 6. Conclusions

- This literature review has shown that agricultural, industrial and postconsumer waste is a promising filler material for composites based on both polymer and metal matrices;
- Its use as reinforcement for composites exhibiting desirable tribological properties only gained in popularity in recent years (especially after 2015);
- The reuse of wastes for tribological applications has advantages, such as environmental and economic benefits, an improvement of mechanical and tribological properties and a reduction in the overall weight of the material;
- Wherever the wear of the produced composites is not worse than the reference materials, their application should be considered for ecological and economic reasons;
- There is a great need to find further wastes that can potentially be useful in improving the tribological properties of the currently used materials;
- Future research should focus not only on the waste that is generated in large quantities but also on that available locally to minimize the environmental impact related to the life cycle of tribological materials.

## Figures and Tables

**Table 1:** Polymer and metal matrix composites reinforced with agricultural waste materials.

Matrix/Mixture		Waste Filler	Reference
Epoxy		*Jatropha curcas* L. fibers	[6,7,8]
		Sugarcane bagasse ash	[9,10]
		Palm kernel fibers	[11,12]
		Pawpaw stem fibers	[13]
		Arhar stalks	[14]
		*Pongamia pinnata* seed cake	[15]
		Bovine femur ash	[16]
		*Phoenix dactylifera* (L.) fruits (dates): date Stones, date flesh	[17]
		Human hair	[18]
		Tasar silk and jute fiber	[19]
		*Citrus limetta* peel	[20]
		Oil palm fruits	[21]
		African land snail shells/mollusk shell	[4]
Polypropylene		Rice husk ash	[22]
		Wood flour	[23]
		Nutraceutical industrial coriander seed spent, and congo red dye adsorbed onto nutraceutical industrial coriander seed	[24]
Polyester		Soap nuts, *Aegle marmelos*, *Terminalia chebula* seeds	[25]
		Bovine hair fibers	[26]
		Palm fronds and mango’s dry leaves	[27]
		Colombian Musaceae	[28]
Recycled waste plastics (type non-specified)		Mussel shells	[29]
Polyvinyl chloride		Olive stone flour	[30]
		Rice husk ash	[31]
Polyethylene		Snail shell	[32]
		Chicken eggshell	[33]
Polyphenylene sulfide		Mussel shells	[34]
Rubber mix		Eggshells, fishbones	[35]
Mixture: silica from Aswan desert; commercial carbon; barium sulfate; metallic powder, phenol–formaldehyde resins		Fibers of corn, palm, and sugar bars	[36]
Mixture: Al_2_O_3_, rock wool, carbon fiber, glass fiber, steel fiber, SiO_2_, MgO, graphite, brass powder, vermiculite, phenolic resin, calcium carbonate and barite		Banana peel, bagasse fiber	[37]
Mixture: vermiculite powder, compound mineral fibers, calcium carbonate, coke, graphite, friction powder, zirconium silicate, alumina, barium sulfate, zinc stearate, phenolic resin		Cornstalk	[38]
Mixture: graphite, coke, zinc stearate, vermiculite powder, calcium carbonate, zirconium silicate, alumina, compound mineral fibers, friction powder, phenolic resin and barium sulfate.		Cow dung fibers, corn stalk fibers	[39]
Mixture: aluminum, graphite, zirconia oxide, silicon carbide, titanium oxide, phenolic resin		Coconut fiber	[40]
Aluminum	AA6351	Rice husk ash	[41]
	pure aluminum		[42]
	A356.2		[43]
	Al-4.5% Cu	Bamboo leaf ash	[44,45]
	Pure aluminum	Eggshell	[2]
	Al-1.5Sn-1.5Mg		[46]
	Al6061		[47]
	AA2014		[48]
	AA1200	Coconut shell ash	[49]
	Al110		[50]
	Al7075		[51]
	Al-Si10-Mg	Sugarcane bagasse ash	[9]
	Al5056		[10]
	A2009	Bean pod ash	[52]
	Al-12% Si	Melon shell ash	[53]
	Al7075	Cow dung ash	[3]
	Al1100	Palm kernel shell ash	[54]
	Al-7Si-0.3Mg	Rice husk ash, fly ash (industrial waste)	[55]
	Al6061		[56]
	A356		[57]
	AZ91D	Eggshell, rice husk ash	[58,59]
	Al6063	Straw ash, met coke ash (industrial waste)	[60]
Zn-ZnO		Snail shell	[61]

**Table 2:** Polymer and metal matrix composites reinforced with industrial waste materials.

Matrix		Waste Filler	Reference
Epoxy resin		Iron mud	[63,64,65,66]
		Red mud	[67,68,69]
		Linz–Donawitz slag	[70,71,72]
		Linz–Donawitz sludge	[72,73,74,75]
		Blast furnace slag	[72,75,76]
		Ferrochromium slag	[76]
		Converter slag	[76]
		Boron wastes	[77,78]
		Glass beads	[79]
		Red brick dust	[80]
		Marble dust	[81]
		Granite dust	[82]
		Coal mine overburden	[83]
		Glass fiber waste and wall tile waste	[84]
		Cenosphere (from power plants)	[85]
		Wastes from hydrometallurgical zinc plant	[86]
Polypropylene		Linz–Donawitz slag	[87]
		Blast furnace slag	[88]
		White aluminum dross	[89]
		E-glass fiber, carbon fibers	[90]
		Iron scale	[91]
		Alumina	[92]
Polyester		Fly ash	[93,94,95]
		Granite dust	[93,95]
		Red mud	[96,97,98]
		Marble dust	[99,100,101]
Polyethylene		Fly ash cenosphere	[102]
Phenolic resin		Slate powder	[103]
Polytetrafluoroethylene		Waste polytetrafluoroethylene	[104]
Polyamide		Biocarbon obtained from the pyrolysis of polymer wastes	[105]
Polyether ether ketone polymer matrix		Recycled carbon fibers	[106]
Aluminum	AA6351	Fly ash	[107]
	Al-Si		[108]
	AA6061		[109]
	Al7075		[110]
	AlLM6		[111]
	Pure aluminum	Red mud	[112]
	Al6061		[113]
	A356	Fly ash and red mud	[114]
	AlLM6	Copper slag	[115]
	Al-Mg-Si	Limestone slurry powder	[116]
	Al7075	Crushed rock sand	[117]
	Pure aluminum	Bottom ash from a pulverized coal combustion boiler	[118]
	AlA356	Furnace steel slag	[119]
	Al6063	Wet grinder stone dust	[120]
	AlA356	Granite particulate	[121]
	AlSi7M	Spent alumina catalyst waste from oil refineries	[122]
	Al-Si	Ceramic particulates	[123]
	AA7075		[124]
Nickel alloy		Recycled tungsten carbide and cobalt hard metal powder	[125]
Silicon bronze alloy		Marble dust	[126]
Copper alloy		Marble dust	[127]

**Table 3:** Polymer and metal matrix composites reinforced with postconsumer waste materials.

Matrix/Mixture	Waste Filler	Reference
Epoxy	Polyethylene bags	[128]
	Waste clothes	[129]
	Porcelain tiles and window glass wastes	[130]
	Tire rubber	[5]
Polypropylene	Tire rubber	[131]
Phenolic resin	Tire rubber	[132]
Polyethylene	Laminated car glass	[133]
Polytetrafluoroethylene	Waste engine oil used as a modifier of polytetrafluoroethylene	[134]
Mixture: polyester, metal fiber, alumina, graphite	Tire rubber, palm slag	[135]
Copper	Cathode ray tube	[136]

## Data Availability

Data sharing not applicable to this article.

## Nomenclature

BF	Blast furnace
CDA	Cow dung ash
COF	Coefficient of friction
FA	Fly ash
HDPE	High-density polyethylene
LD	Linz–Donawitz
PA	Polyamide
PE	Polyethylene
PEEK	Polyether ether ketone
PP	Polypropylene
PTFE	Polytetrafluoroethylene
RM	Red mud
RHA	Rice husk ash
WTD	Waste tire dust
